# Secret Voices are Breaking the Silence: A Meta-Ethnography of Perceptions of Sexual and Reproductive Health Among Resettled Refugee Youth

**DOI:** 10.1177/23333936251330688

**Published:** 2025-04-30

**Authors:** Tone Hjelm, Terese Bondas, Bente Kristin Høgmo

**Affiliations:** 1 Stavanger Municipality, Norway; 2 University of Stavanger, Norway

**Keywords:** refugee, youth, sexual and reproductive health, nursing, perception, knowledge, experience, attitude, meta-ethnography

## Abstract

Nurses are in the front line in caring for refugee youth in relation to their sexual and reproductive health. Culturally competent nursing care requires an understanding of different health values, beliefs, and practices and to be aware of the perceptions refugee youth have regarding sexual health. Therefor the aim of this meta-ethnography was to synthesize knowledge of refugee youth, and their perceptions related to sexual and reproductive health, before and after resettlement to a new country. Nine qualitative studies were included, describing the experiences of 297 refugee youth, originating from 13 countries, resettled in respectively Australia, USA and in temporary resettlements in Lebanon. *Secret voices are breaking the silence* was established as an overarching metaphor in a lines-of-argument synthesis. This metaphor was accompanied by three main themes: (1) *The sounds of silence;* (2) *We have no words for it;* and (3) *Longing to learn*. Findings indicate that cultural values and beliefs represent a barrier for refugee youth in accessing sexual and reproductive health information, services, and care. In a resettlement context language is a barrier to access sexual health information, and fear of judgment from family, friends, and community holds young refugees back from seeking services and care. Young refugees are longing for more knowledge, for themselves and their parents. This meta-ethnography can contribute to a heightened awareness amongst nurses in providing sensitive and culturally competent care for a diverse population of refugee youth.

## Introduction

Globally, there is convincing evidence that sexual and reproductive health information has a positive effect on adolescents’ sexual health and behavior (Chandra-Mouli et al., 2015; World Health Organization [WHO], 2018). Information enables young people to advocate for their health, well-being, and dignity (Chandra-Mouli et al., 2015). Sexual and reproductive health and rights for adolescents and young people were first introduced in 1994 at the *International Conference on Population and Development* (Ruzibiza et al., 2021). In 2012, *United Nations Commission on Population and Development* emphasized young people’s right to access sexual and reproductive health information and services, and to be able to decide freely on matters related to their sexuality (Chandra-Mouli et al., 2015). Yet, research shows that most adolescents lack knowledge of sexual and reproductive health, leaving them vulnerable to sexual coercion, sexually transmitted infections, and unintended pregnancies (United Nations Population Fund, 2021). *The 2030 Agenda for Sustainable Development* (United Nations, 2024) emphasizes the importance of universal access to sexual and reproductive health care and rights for young people.

Young refugees are seen as a particularly vulnerable group, at risk of not having their rights fulfilled (Starrs et al., 2018). The barriers in accessing sexual and reproductive health services are exacerbated in a refugee context, whereas simultaneously there is a higher risk of experiencing sexual and gender-based violence (Bukuluki et al., 2021; Tirado et al., 2020). Girls are at risk of forced/child marriage, sexual abuse, and sex trafficking, while boys are equally at risk of experiencing sexual abuse and sex trafficking (Mason-Jones & Nicholson, 2018; Starrs et al., 2018, p. 2670). Refugee youth further experience several barriers to access sexual health information and care, both before and after resettlement to a new country (Lirios et al., 2024; Napier-Raman et al., 2023). As sexual and reproductive health is best understood in the context of a wide range of beliefs, practices, behaviors, and identities (UNESCO, UN Women, UNICEF, UNFPA, & Joint United Nations Program on HIV/AIDS, 2018), it is pivotal for nurses to recognize how cultural values and beliefs exert an influence on young refugees’ perceptions related to sexual and reproductive health. This knowledge can support nurses in providing culturally competent health care.

The terms *refugee youth* and *young refugees* will be used interchangeably. The term “youth” is defined by the United Nations as those between 15 and 24 years, and “young people” as those between 10 and 24 years, a term used by the World Health Organization and others to combine adolescents and youth (WHO, 2023). Refugees are people who have fled war, violence, conflict, or persecution, and have crossed an international border to find safety in another country (UN High Commissioner for Refugees (UNHCR), 2023). Whenever the term *sexual health* is used, our intention is that it comprises both the term *sexual* and *reproductive* health.

## Background

Being forced to migrate has a negative impact on one’s bodily autonomy (Tirado et al., 2020) and the ability to negotiate safe sex (Chandra-Mouli et al., 2015; Ruzibiza et al., 2021). Chandra-Mouli et al. (2015) report that adolescents are more likely to experience sexual and reproductive health needs, but less likely to utilize health services due to structural challenges, such as poverty and homelessness. Underutilization of sexual and reproductive health services is common in refugee camps (Kwankye et al., 2021). Furthermore, being forced to flee disrupts family and social relations, and youth-friendly services are often distant or absent (Bukuluki et al., 2021; Ruzibiza et al., 2021). Studies show that refugee youth have a limited (Chandra-Mouli et al., 2015), inaccurate or no knowledge of sexual and reproductive health and rights (Aibangbee et al., 2023; Napier-Raman et al., 2023; Tirado et al., 2020). They receive little information about safe sex and contraception use, information is often provided too late, after having contracted a sexually transmitted infection or becoming pregnant (Lirios et al., 2024; Tirado et al., 2020). They do not see themselves at risk, consequently they do not access health services. Young women hold misconceptions of abortions due to the knowledge of unsafe abortion procedures in their country of origin (Chandra-Mouli et al., 2015).

Young refugees who are resettled in individualistic societies can experience conflicts between their own traditional norms and values, and the liberal values of the host country (Lirios et al., 2024; Tirado et al., 2020). Some experience difficulties in talking with their parents on matters concerning sexual health (Kwankye et al., 2021; Lirios et al., 2024), and diverse cultural beliefs concerning sexual health can lead to intergenerational conflicts (Tirado et al., 2020). Refugee youth can also feel pressured to conform to norms of their peers and stereotypes produced by mass media in the host country, and a lack of judgment can lead to risky behavior (Chandra-Mouli et al., 2015). In a resettlement context where the social and legal context is new and unfamiliar, sexual, and reproductive health is often not a priority. Accessing services and care can additionally be undermined due to perceived stereotypical judgment from health professionals (Aibangbee et al., 2023; Tirado et al., 2020) and language barriers (Kwankye et al., 2021).

Available studies report on the needs refugee youth have concerning sexual and reproductive health and the barriers they face both pre- and post-resettlement. However, the knowledge of refugee youth and their perceptions regarding sexual and reproductive health in a resettlement context seem to be limited to individual qualitative studies. Toward the background of an increase in the global refugee situation, there is a need to synthesize available knowledge across qualitative studies from different countries to help increase understanding amongst nurses, and to have an impact on health promotion and prevention.

## Aim and Research Question

The aim of this meta-ethnography was to synthesize knowledge of refugee youth, and their perceptions related to sexual and reproductive health, both before and after resettlement to a new country. The goal is to increase the knowledge base, and the awareness among nurses and health organizations, on which barriers refugee youth face in accessing sexual and reproductive health information, services, and care. This can raise the awareness of nurses, and ensure equitable sexual and reproductive health information, services, and care for refugee youth. It is also important to the awareness of young refugees themselves and their relatives in a wider societal perspective. The research question therefore is: Which perceptions do refugee youth have related to sexual and reproductive health?

## Methods

Meta-ethnography (Noblit & Hare, 1988) was chosen to synthesize the findings from the individual qualitative studies. We chose this qualitative synthesis approach as it is interpretative, using Geertz’ concept of thick description and Turner’s theory of understanding as translation (Noblit & Hare, 1988). Meta-ethnography is an effective and suitable method for developing and presenting new models and theories that go beyond the findings from individual studies (France et al., 2019). Meta-ethnography is appropriate to use, especially when doing research on sensitive topics like sexual and reproductive health (Bondas et al., 2021, p. 12; France et al., 2019). Integrating and synthesizing research from individual studies to acquire new insights is in accordance with the United Nations (2024) sustainability goals. To prevent research waste, one should avoid conducting new data collection when available knowledge exists, especially on ethically sensitive topics.

This study follows Noblit and Hare’s (1988) method, consisting of seven overlapping phases, as a nonlinear interpretive approach. The phases are: (1) Getting started, (2) deciding what is relevant, (3) reading the studies, (4) determining how the studies are related, (5) translating the studies into one another, (6) synthesizing translations, and finally, (7) expressing the synthesis. Moreover, the eMERGe reporting guidance developed by France et al. (2019) was used to improve the clarity and integrity of meta-ethnographic reporting.

## Data Collection and Analysis

### Phase 1: Getting started

Sexual and reproductive health and rights for refugee youth and equitable health services are topics of great engagement and importance for all authors. We are public health nurses by education. The first author (TH) is currently working at a youth health center and at a school for young refugees and migrants. The second and third authors (TEB and BKH) both have professional experience in caring for refugees and doing research on topics related to youth and migrant health in Scandinavia. We have had several discussions and reflected upon our own cultural background when conducting research on a refugee population, and ethical considerations on how to avoid generalization that can reinforce a stereotypical view. Our common academic and professional interest in sexual and reproductive health information and care aroused our curiosity on where refugee youth get information about sexual and reproductive health, and their own perception concerning this topic. Two of the authors have extensive and varied experience as qualitative researchers, including meta-ethnography. Ethics approval was not required for this meta-ethnography.

### Phase 2: Deciding What is Relevant

We developed the inclusion and exclusion criteria to allow for a specific, systematic, and thorough search. The inclusion and exclusion criteria (see Table 1) were refined as the search process progressed.

**Table 1.** Inclusion and Exclusion Criteria.

Inclusion	Exclusion
• Females and males aged 10 to 25	• Females and males over the age of 25
• Refugees	• Migrant, asylum seeker, children born in the host country of immigrants and refugee parents
• English and Scandinavian languages	• Studies written in other languages than English or Scandinavian
• Studies published after 2005	• Studies published before 2005
• Peer-reviewed primary qualitative studies. Studies that use mixed methods when it is possible to distinguish between qualitative and quantitative findings.	• Quantitative studies
• Sexual and reproductive health, i.e., studies focusing on knowledge, experience and attitudes.	• Studies on one topic, e.g., sexually transmitted infections, contraception, female genital mutilation, gender-based violence
• Resettlement context and refugee camps	

**Figure 1.**
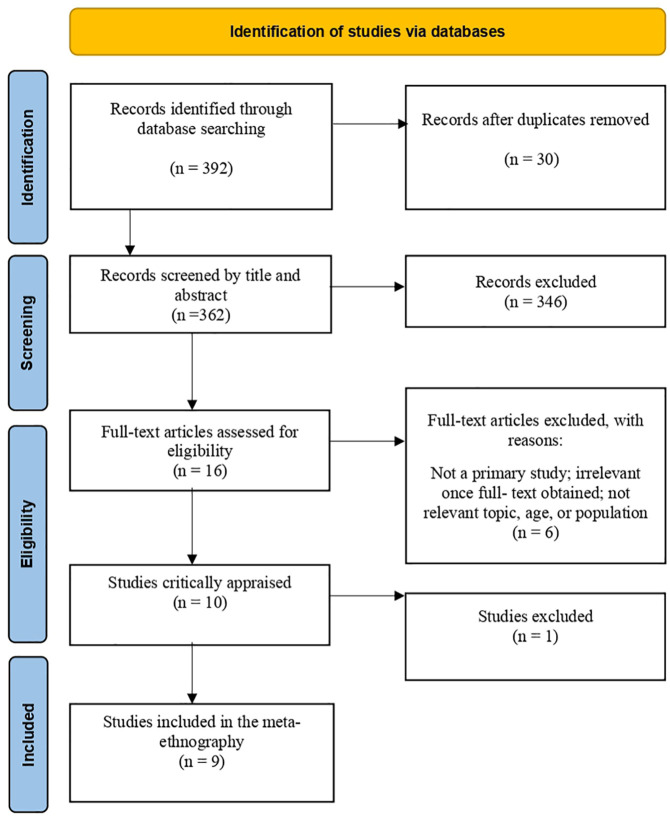
An adapted PRISMA flow-chart of the literature search (Page et al., 2021).

The inclusion criteria for the population were initially young girls and boys between 10 and 19. Limited research was found on young adolescents: the upper age limit was therefore changed to 25, a range informed by the included studies. Migrants were excluded as this group might consist of individuals who were born and raised in a country and hence might have acquired knowledge of sexual health and services. Asylum seekers were excluded because this group is awaiting a decision on their application for international protection. During the literature search process, the search limiters were set to exclude studies published before 2010, consequently excluding nine studies. However, numerous research papers cited an Australian study that seemed relevant. To include this study, the year limit was therefore changed to 2005. The search strategy was developed together with a university librarian. The following search terms were applied: *refugee* [and] *adolescent* [or] *young people* [or] *youth* [or] *teen* [or] *young adults* [and], *sexual health*. Initially, the terms *knowledge*, *experience* and *attitudes* were included. But by including these, whether alone or in combination with similar words, for example, *understanding, awareness, information, belief and attitude*, the search results yielded fewer or non-relevant hits. A literature search was conducted in databases relevant to the review question: CINAHL, ERIC, MEDLINE, SocINDEX TRC, and Scopus, with a cut-off in February 2022. An updated search was performed by the first (TH) and third author (BKH) in January 2024, including a forward citation search in Google Scholar. A manual search was made in the relevant websites, organizations, research institutions, and reference lists of the included studies. We used the PRISMA flowchart (Page et al., 2021) to record the selection process (Figure 1).

Out of 392 records identified through database searches, 30 duplicates were removed. A total of 362 records were screened by title and abstract, with six full-text articles excluded, according to inclusion and exclusion criteria. Ten studies were eligible to be critically appraised according to the Critical Appraisal Skills Program (CASP), a checklist for qualitative research (2018; see Table 2). One study was excluded, according to criteria *#3: Was the research design appropriate to address the aims of the research?* The researchers themselves noted that the interview guide had been designed in a way that did not directly affect the topics of interest, namely, to improve acceptability among the study population (Ortiz-Echevarria et al., 2017).

**Table 2.** Critical Appraisal of Included Studies (CASP).

Critical appraisal questions	1	2	3	4	5	6	7	8	9	10
Article										
McMichael and Gifford (2009)	Y	Y	Y	Y	Y	C	Y	Y	Y	Y
McMichael and Gifford (2010)	Y	Y	Y	Y	Y	C	Y	Y	Y	Y
Dhar et al. (2017)	Y	Y	Y	Y	Y	N	C	Y	Y	Y
Dean et al. (2017)	Y	Y	Y	Y	Y	C	Y	Y	Y	Y
Ortiz-Echevarria et al. (2017)	Y	Y	N	Y	N	Y	Y	Y	C	N
Kingori et al. (2018)	Y	Y	C	C	Y	C	C	Y	Y	Y
Kaczkowski and Swartout (2020)	Y	Y	Y	Y	Y	C	C	Y	Y	Y
El Ayoubi et al. (2021)	Y	Y	Y	Y	Y	N	Y	Y	Y	Y
Korri et al. (2021)	Y	Y	Y	Y	Y	C	C	Y	Y	Y
Kumar et al. (2021)	Y	Y	Y	Y	Y	C	Y	Y	Y	Y

*Note.* Critical appraisal questions: (1) Was there a clear statement of the aims of the research? (2) Is a qualitative methodology appropriate? (3) Was the research design appropriate to address the aims of the research? (4) Was the recruitment strategy appropriate to the aims of the research? (5) Was the data collected in a way that addressed the research issue? (6) Has the relationship between researcher and participants been adequately considered? (7) Have ethical issues been taken into consideration? (8) Was the data analysis sufficiently rigorous? (9) Is there a clear statement of findings? (10) How valuable is the research? Y = yes, N = no, C = can’t tell.

### Phase 3: Reading the Studies

This phase, managed by the first author (TH), involved a repetitive reading of the studies. The co-authors focused on the findings of the studies. During the reading phase, an identification of the characteristics of the studies were done. Notes were taken on a preliminary identification of possible concepts, metaphors, and themes, while looking for the similarities and differences between them (Noblit & Hare, 1988). It was a movement of going back and forth within- and between the studies (France et al., 2019). Guided by the review question, the studies were searched for content that could give meaning to the perceptions the study participants had regarding sexual and reproductive health, paying attention to what each account revealed (Noblit & Hare, 1988). An overview of the included studies and its characteristics is presented in Table 3. Two studies shared the same study population and were conducted by the same researchers (McMichael & Gifford, 2009, 2010).

**Table 3.** Characteristics of Included Studies.

First author	Country/context	Design/Method	Aim/purpose	Participants	Age	Country of origin	Results
McMichael and Gifford (2009)	Melbourne, Australia	Semi-structured in-depth interviews	To identify how young people with refugee backgrounds access, interpret and implement information about sex and sexual health	n = 142 75 females 67 males	16–25	Afghanistan	Young people with refugee background had little knowledge of sexual health or STIs apart from HIV/AIDS. While they are aware of potential sources of sexual health information, few of these sources are utilized. Specific barriers to learning about sexual health include concerns about confidentiality, shame and embarrassment when discussing sexual health, and the competing demands of resettlement.
	Post-settlement context	Focus group discussions				Burma/Myanmar	
						Ethiopia	
						Horn of Africa-countries	
						Iraq	
						Liberia	
						Sudan	
McMichael and Gifford (2010)	Melbourne, Australia	Semi-structured in-depth interviews	To explore how resettled youth access, interpret and implement sexual health information, with a particular focus on how social contexts shape attitudes and understandings	n = 142 75 females 67 males	16–25	Afghanistan	Young people had some knowledge of HIV and AIDS, knowledge of other STIs was limited. Importantly, narratives about risk and protection were informed by concerns for maintenance of social wellbeing.
	Post-settlement context					Burma	
						Ethiopia	
						Horn of Africa-countries	
						Iraq	
						Liberia	
						Sudan	
Dean et al. (2017)	Brisbane, Logan and South-East Queensland, Australia	Semi-structured face-to-face interviews, thematic analysis	To develop intergenerational understanding of the factors perceived to be influencing the sexual health and wellbeing of young Sudanese refugees in Queensland, Australia	n = 11 males	16–24	Sudan	Sexual health-related knowledge, attitudes, and beliefs, along with patterns of sexual behavior, are changing post-resettlement and this create considerable intergenerational discord and family conflict
	Post-settlement context						
Dhar et al. (2017)	Philadelphia, USA	Semi-structured individual interviews	To explore attitudes and beliefs pertaining to sexual and reproductive health (SRH) among unmarried, female, resettled Bhutanese refugees	n = 14 females unmarried no children	16–20	Bhutan	SRH was stigmatized for unmarried youth, making seeking information about SRH or accessing family planning difficult. There were many misconceptions about access to SRH.
	Constant comparative method	Post-settlement context					
Kingori et al. (2018)	Ohio, USA	Semi-structured in-depth interviews	To identify sexual health knowledge barriers among Somali young adults in Ohio	n = 27 14 females 13 males	18–25	Somalia	Findings revealed sexual health knowledge barriers in the following broad categories: religion, culture and stigma. Cultural and religious norms were deemed an important influence on the community norms largely impacting sexual health knowledge due to stigma and fear of judgment. Participants overcame barriers by seeking information from external sources such as doctors, Internet, and peers
	Post-settlement context						

Kaczkowski and Swartout (2020)	Atlanta, USA	Semi-structured in-depth interviews	The purpose is to understand sexual health literacy, sources and barriers to access across groups of resettled refugee men and women	n = 25 12 women 13 men Unmarried No children	18–24	Afghanistan	Both men and women appeared to have limited knowledge about sexual health. School was their primary source of information; women also talked with their parents, whereas men preferred to reach out to teachers, peers and online sources. For both groups, barriers to access included language difficulties and lack of money, insurance and transport. Men also stressed concerns about confidentiality, whereas women focused on shame and embarrassment when discussing sexual health
	Post-settlement context	Focus group interviews				Burma/Myanmar	
		Thematic coding				Central African-Republic	
						Colombia	
						DR Congo	
						Pakistan	
						Somalia	
El Ayoubi et al. (2021)	Lebanon’s Bekaa governorate on the border to Syria	Focus group discussions	The aim of the present study was threefold: (a) to understand what information Syrian refugee adolescent girls in Lebanon receive about puberty and SRH; (b) to discern how this information shapes their experiences as they transition to adulthood; and (c) to identify the sources of the information received	n = 26 females unmarried	14–20	Syria	Our findings highlighted that adolescent participants received inadequate SRH information shortly before or at the time of menarche and sexual initiation, resulting in experiences characterized by anxiety and fear
	Thematic analysis (Braun and Clarke)	Displacement context					
Korri et al. (2021)	Bourj	Semi-structured in-depth interviews	To learn about the SRH perceptions and experiences of refugee adolescent girls living in Bourj Hammoud, an urban setting in Lebanon	n = 40 females	13–17	Syria (Arab and Kurdish)	The majority of the FGD participants reported a lack of awareness about menstruation when they experienced it for the first time and the social stigma associated with menstruation. When defining puberty, they indicated its social link to a girl’s readiness for marriage and her need to become cautious about sexual harassment. Most FGD participants had very poor knowledge of the female reproductive system. Mothers were the most approached persons to receive information on SRH issues; however, the girls indicated a wish to receive advice from specialists in a comfortable and private atmosphere. All the girls reported that either they themselves, or an acquaintance, had experienced some type of sexual harassment. The girls rarely reported those incidents due to fear of being blamed or subjected to mobility restrictions or forced to drop out of school.
	Hammoud	Focus group discussions					
	Urban area of Beirut, Lebanon						
	Displacement context						
Kumar et al. (2021)	Large southeastern city, USA	Semi-structured interviews	The purpose of this study was to identify barriers and facilitators to refugee young women’s sexual health across multiple systems and how factors within these systems intersect to influence sexual health behavior.	n = 12 females	18–24	Burma	Findings revealed four primary themes: sex/relationship restrictions, judgment/disapproval, support, and youth outcomes. Themes varied by context (e.g., family, peers, religion, culture) and were related to one another in important ways, such that refugee young women who violate sociocultural sex/ relationship expectations experience actual or anticipated judgment from others, which leads to fear, embarrassment, and risky sexual behavior.
	Post-settlement context	Thematic text analysis				Central African-Republic	
		Narrative analysis				DR Congo	
						Somalia	

### Phase 4: Determining How the Studies are Related

After repeated readings of the studies and discussions between all three authors, the findings were juxtaposed to determine how they were related. According to Noblit and Hare (1988), the relationship between the studies appears as either reciprocal (similar), refutational (in opposition) or in line-of-argument (cumulative). After the preliminary analysis, the findings were found to be analogous and an assumption of a reciprocal relationship between the studies was made (Noblit & Hare, 1988).

### Phase 5: Translating the Studies into One Another

The translation process is an interpretative process that is idiomatic rather than literal. An interpretation of *meaning* is the key (Noblit & Hare, 1988), which implies looking for the possible meaning of quotes and findings. Translations preserve the central concepts of each study, and hence conserve the meaning of each concept (Noblit & Hare, 1988). This is what distinguishes meta-ethnography from other qualitative evidence syntheses (France et al., 2019, p. 2). We chose an index article that contained rich data (McMichael & Gifford, 2009). We extracted concepts, metaphors and phrases (Noblit & Hare, 1988) from the index article, being open and reflective while moving on to the next article. During the extraction process, we attempted to see whether there was a relationship between the concepts and the studies, looking for commonalities, overarching concepts, or metaphors, and noting preliminary interpretations (France et al., 2019). We then did a step-by-step process of translating the studies into one another, first by comparing the meaning of concepts within the same study, then proceeding in the same manner to the next study. The concepts with shared meaning were color-coded. The translation process is exemplified in Table 4 (see Supplemental File for the translation process). The extracted data was derived from both participants’ quotations, and the original authors’ concepts, also referred to as *first and- second order constructs* (Noblit & Hare, 1988). France et al. (2019, p. 7) argue that it is not apparent how these are distinguished, as authors typically choose quotes that support their analysis. Therefore, first- and second-order constructs can be analyzed and synthesized together, though not independently. In the matrix, the participant’s quotation was kept track of by using quotation marks. The translation process enabled the findings to be coded, eventually being organized into themes and an integrated synthesis.

### Phase 6: Synthesizing Translations

After finalizing the process of translation, the extracted themes and metaphors were analyzed. This process gave rise to a new interpretation or overarching metaphor, going beyond the findings of each of the individual studies (Noblit & Hare, 1988), and three themes were developed as shown in Table 5 (see Supplemental File for the translation to sub-themes, themes, and overarching metaphor). Throughout the interpretive process, all the authors discussed the themes and metaphors, and reflected upon the occurrent pre-understanding, thus enabling new perspectives and interpretations. Guided by Noblit and Hare (1988), and the guidance by France et al. (2019), the translation proceeded from reciprocal translation to a lines-of-argument synthesis (France et al., 2019; Noblit & Hare, 1988). The synthesis refers to making a whole into something more than the parts alone imply (Noblit & Hare, 1988). However, we also noted refutations in the findings (Noblit & Hare, 1988) regarding the perceptions of sex before marriage in the studies. Some young refugees chose to have sexual relationships before marriage, while others shared that they would like to wait. Still, the findings were comparable and analog. The studies taken together represent a new understanding, a *metaphorical lines-of-argument synthesis to create understanding* that goes beyond the original studies (Noblit & Hare, 1988). Supplemental Figure shows the lines-of-arguments synthesis.

### Phase 7: Expressing the Synthesis

The meta-ethnography is presented as a scientific article including the metaphorical lines-of-arguments synthesis based on three themes and sub-themes, but could also be presented in other ways, for example, more popular or artistic presentations (Noblit & Hare, 1988). To ensure that the synthesis is expressed in a way that is relevant to the audience, the chosen language and concepts were used to enrich the human discourse by displaying the phenomena from new and multiple perspectives (Noblit & Hare, 1988).

## Findings

The young refugees in this meta-ethnography came from the following countries: Afghanistan, Bhutan, Burma/Myanmar, Central African Republic, Colombia, the Democratic Republic of Congo, Ethiopia, Iraq, Liberia, Pakistan, Somalia, Sudan, and Syria. Most of them had lived in the country of resettlement for 1 to 5 years. Although recognizing the heterogeneity of these groups of young refugees, patterns of experiences are visible in the studies. In the refugee context refugee youth experienced broken social and family networks, disrupted education, barriers to sexual health information and access to health services. Additionally cultural and religious beliefs and disbeliefs presented a barrier to information both before and after resettlement. In the resettlement context, a new language, limited knowledge of sexual and reproductive health and rights and fear of judgment presented barriers to accessing sexual health information and care (see Supplemental File for more details regarding context). The studies included in this meta-ethnography are within a range of 12 years, yet there were no significant differences in the findings.

*Secret voices are breaking the silence* was identified as an overarching metaphor in a lines-of-arguments synthesis. The analysis of interpretative reciprocal findings of the translation process resulted in three main themes: (1) The sounds of silence; (2) We have no words for it; and (3) Longing to learn.

## The Sounds of Silence

The first theme, *“The sounds of silence”* consists of three subthemes: (a) A culture of silenced shame; (b) Sex is only a word until marriage; and (c) Nowhere to learn.

### A Culture of Silenced Shame

In several studies, young refugees experienced a silence from their surroundings concerning sexual and reproductive health. Shame stood out as a powerful barrier to sexual health knowledge, and communication about non-marital sex (Dean et al., 2017; Dhar et al., 2017; El Ayoubi et al., 2021; Kingori et al., 2018; Korri et al., 2021; Kumar et al., 2021; McMichael & Gifford, 2009). If young women were perceived to behave poorly, they brought shame on themselves, their families, and their communities (Dean et al., 2017). One female participant said, “She can’t face anyone in our culture because she makes shame on it” (McMichael & Gifford, 2010, p. 269). The stigma attached to non-marital pregnancy was such that it could coerce young women into marriage (Dhar et al., 2017). Parents warned the youth about the consequences a non-marital pregnancy could have on the reputation of the individual and the family (McMichael & Gifford, 2009). Fear of judgment from the community (Kingori et al., 2018) could contribute to unhealthy behavior among young female refugees, such as avoiding getting tested for sexually transmitted infections (Kumar et al., 2021). Refugee youth would also be hesitant to reach out to their peers out of fear of judgment (2021), and that they would disclose the information to others (McMichael & Gifford, 2009). In a new country where the communities are small, youth had concerns seeking sexual health services in case they were seen by community members (Kaczkowski & Swartout, 2020; Kumar et al., 2021; McMichael & Gifford, 2009). They were also scared that interpreters would spread information (McMichael & Gifford, 2009). If the community found out about non-marital relationships- and pregnancies, the youth experienced they would be laughed at, and people would think bad of them (Dhar et al., 2017; Kumar et al., 2021). A female participant illustrated the fear of judgment by saying, “How to go to a supermarket and ask for a condom [. . .] people don’t have that freedom” (Kumar et al., 2021, p. 1800). The lack of an open discussion contributed to a generalized silence, and caused a delay in information (Dean et al., 2017; El Ayoubi et al., 2021).

### Sex is Only a word until Marriage

The cultural non-acceptance of sexual relationships before marriage was brought up in several studies (Dean et al., 2017; Dhar et al., 2017; Kumar et al., 2021; McMichael & Gifford, 2009). Parents would not permit relationships prior to marriage and were not willing to speak with their children about it (Dean et al., 2017; Kumar et al., 2021). Opposing one’s parents could have grave consequences. Being involved in an unsanctioned romantic relationship might result in being kicked out of the home (McMichael & Gifford, 2009) or punished if their parents found out (Kumar et al., 2021). A female participant shared, “If I went to my mum with that information, I am dead” (Kingori et al., 2018, p. 344). Abstinence from sex before marriage was emphasized as a cultural value that the young refugees were expected to uphold (Dean et al., 2017; Dhar et al., 2017; Kaczkowski & Swartout, 2020; Kumar et al., 2021; McMichael & Gifford, 2010). The focus on abstinence before marriage shaped young refugees’ view on contraception, as the use of contraception was primarily thought of as a means to avoid pregnancy, and less as protection against sexually transmitted infections (Dean et al., 2017; Dhar et al., 2017; El Ayoubi et al., 2021; McMichael & Gifford, 2010).

### Nowhere to Learn

Several studies noted that learning opportunities for refugee youth was limited (El Ayoubi et al., 2021; Kingori et al., 2018; Korri et al., 2021; Kumar et al., 2021; McMichael & Gifford, 2009, 2010). Some youth experienced not learning about sexual and reproductive health at school (McMichael & Gifford, 2009). Some were excluded from sexual health education because of language barriers (Dhar et al., 2017). Others reported that parents kept them home on the days of sexual health education, on religious leaders’ advice (Kingori et al., 2018). Learning about sexual health before marriage could encourage sex, thereby violating cultural norms (Dean et al., 2017; Kaczkowski & Swartout, 2020). A female participant shared: “If I don’t have any husband or am not married yet, I don’t have any idea who I could talk to” (McMichael & Gifford, 2009, p. 227). All studies reported that knowledge about sexual and reproductive health among refugee youth was limited (Dean et al., 2017; Dhar et al., 2017; El Ayoubi et al., 2021; Kaczkowski & Swartout, 2020; Korri et al., 2021; Kumar et al., 2021; McMichael & Gifford, 2009, 2010). They held misconceptions and a lack of knowledge of sexually transmitted infections (Dean et al., 2017; Kaczkowski & Swartout, 2020; McMichael & Gifford, 2010), contraception (Dean et al., 2017; El Ayoubi et al., 2021; Kaczkowski & Swartout, 2020), menstruation and the female reproductive system (El Ayoubi et al., 2021; Korri et al., 2021), as well as laws pertaining to sexual and reproductive health and rights (Dhar et al., 2017; Kaczkowski & Swartout, 2020). In several studies, mothers were cited as a trusted source of information, especially for female participants (El Ayoubi et al., 2021; Kaczkowski & Swartout, 2020; Korri et al., 2021; Kumar et al., 2021; McMichael & Gifford, 2009). However, mothers’ messages were limited to child rearing (McMichael & Gifford, 2009), abstinence from sex (Kumar et al., 2021), religious injunctions and obedience to their husbands (El Ayoubi et al., 2021). For some refugee youth, a new language posed a challenge, thereby making it difficult to find out about laws on confidential health services (Dhar et al., 2017), and access sexual and reproductive health services and care (McMichael & Gifford, 2009).

## We Have No Words for It

The second theme, *“We have no words for”* consists of two subthemes: (a) We can’t have that talk and (b) What is to come is unknown.

### We Can’t Have That Talk

Living in an environment where not talking about sexual health was the norm, manifested itself in an avoidance in seeking care and information, due to embarrassment (Kaczkowski & Swartout, 2020). Several studies underlined that sexual and reproductive health was considered a sensitive topic that brought up a lot of embarrassment for refugee youth (El Ayoubi et al., 2021; Kaczkowski & Swartout, 2020; Korri et al., 2021; McMichael & Gifford, 2009), thus hindering daughters in talking with their mothers (El Ayoubi et al., 2021; Korri et al., 2021). Talking with parents about sex also caused fear (Kumar et al., 2021) and concerns that parents would mistake them for being interested in someone (Kaczkowski & Swartout, 2020). Discussing sensitive matters with health professionals could be difficult (McMichael & Gifford, 2009). If they were asked too many questions about sex and relationships it could be perceived as intrusive and might cause the youth not to return (Kaczkowski & Swartout, 2020). Concerns regarding confidentiality could also prevent young refugees from seeking health services (Kaczkowski & Swartout, 2020; McMichael & Gifford, 2009). Religion was seen as a significant barrier to acquire knowledge, and brought about fear in people (Kingori et al., 2018).

### What is to Come is Unknown

A lack of communication concerning sexual and reproductive health meant that the young refugees were not prepared. Not having knowledge about menstruation caused shock, which led to negative emotions around menstruation (Korri et al., 2021). Female participants were worried and scared about sexual intercourse (Korri et al., 2021; McMichael & Gifford, 2010). The contrast between marriage expectations and real-life experiences was notable (El Ayoubi et al., 2021; McMichael & Gifford, 2009). A female participant was told by her husband on their wedding night: “Girls who are not calm and who move [during sex] end up going to the hospital,” saying that sex would hurt, but that is normal (El Ayoubi et al., 2021, p. 989). A friend of a female participant was told by her mum to not scream; when she woke up the next morning, she knew what her mum had meant (McMichael & Gifford, 2009, p. 227). Memories from the wedding night brought up the fear, worry and loneliness that they had felt that night. They wished they had received advice about the sexual, emotional, and intimate aspect of marriage before it took place, with a female participant stating: “How a husband should treat a wife” (El Ayoubi et al., 2021, p. 993). Some of the participants had internalized the view on not discussing matters of sexuality (Kingori et al., 2018). When parents told them that it is not the right time to learn about sex, they trusted their parent’s judgment (Kaczkowski & Swartout, 2020).

## Longing to Learn

The third theme, “*Longing to learn*” consists of three subthemes: (a) A desire to learn; (b) Knowledge is to be found; and (c) The young people are out there.

### A Desire to Learn

It is not the young refugees’ personal attitudes that stand in the way of learning about sexual and reproductive health, but rather the contextual and structural challenges they encounter (McMichael & Gifford, 2009). The young refugees in several of the studies showed an interest and curiosity in learning more about sexual and reproductive health (Dean et al., 2017; Dhar et al., 2017; El Ayoubi et al., 2021; Kaczkowski & Swartout, 2020; Kingori et al., 2018; Korri et al., 2021; McMichael & Gifford, 2009). A male participant said, “It’s good to know now (. . .) before it’s too late” (McMichael & Gifford, 2009, p. 225). They realized they had limited knowledge, which often meant learning the hard way; by experience (McMichael & Gifford, 2010). Participants talked about secret relationships, their primary concern was to make sure their parents would not find out (Kaczkowski & Swartout, 2020; Kumar et al., 2021; McMichael & Gifford, 2009), as they were ashamed of breaking parental rules (Kumar et al., 2021). Avoiding physical contact was regarded as difficult for some (McMichael & Gifford, 2010). A male participant stated: “You think you can avoid sex (. . .) there’s no way to avoid sex” (Kaczkowski & Swartout, 2020, p. 375). Some youth shared the importance of taking responsibilities for their own choices and health (Kaczkowski & Swartout, 2020), which implied getting tested before engaging in sexual activities (Kaczkowski & Swartout, 2020; McMichael & Gifford, 2010).

### Knowledge is to be Found

A source of information about sexual health that was frequently cited by the youth was friends and social network (El Ayoubi et al., 2021; Kaczkowski & Swartout, 2020; McMichael & Gifford, 2009). Male participants felt at ease when discussing sexual health with friends they could relate to (Kaczkowski & Swartout, 2020). Female participants reported that receiving information from other female members of the family made them view menstruation as normal (El Ayoubi et al., 2021), and they accessed female family members if they needed help (Korri et al., 2021). The internet was another frequent source of information, especially among males (Kaczkowski & Swartout, 2020; Kingori et al., 2018; McMichael & Gifford, 2009). The internet was viewed as a source of independence and autonomy, simplified using mobile phones and Google (Kingori et al., 2018). Schools were seen as an appropriate and credible place for learning about sexual and reproductive health (Dean et al., 2017; Dhar et al., 2017; Kingori et al., 2018; McMichael & Gifford, 2009), providing a confidential space for unmarried youth (Dhar et al., 2017). Youth considered school-based sexuality education as valuable and informative (McMichael & Gifford, 2009). A quote from a female participant signifies the importance of school-based sexuality education: “I learned everything from school” (Kingori et al., 2018, p. 345). Youth shared that they would love to talk to someone who knows, someone with experience in the field of sexual and reproductive health (Kaczkowski & Swartout, 2020; Korri et al., 2021; McMichael & Gifford, 2009). They wanted information in an understandable manner from health professionals (Kaczkowski & Swartout, 2020; McMichael & Gifford, 2009).

### The Young People are out There

Participants emphasized the need for community outreach and information about free sexual and reproductive health services (Kaczkowski & Swartout, 2020; Kumar et al., 2021). One female participant urged the health clinics to reach out by saying: “The young people are out there” (Kumar et al., 2021, p. 1798). Youth preferred to receive information from someone they could confide in (El Ayoubi et al., 2021; Kaczkowski & Swartout, 2020; Kingori et al., 2018). In one study, males emphasized discretion and trustworthiness, and females’ empathy and personal rapport (Kaczkowski & Swartout, 2020). They expressed that they needed knowledgeable staff who would alleviate feelings of shame and embarrassment (Kaczkowski & Swartout, 2020), and who were encouraging and non-judgmental (Kumar et al., 2021). A female participant expressed this by saying: “It’s all about making someone feel like it’s ok” (Kaczkowski & Swartout, 2020, p. 376). An important concern for refugee youth was that parents acquired a heightened awareness of sexual and reproductive health, and the value of information (Dean et al., 2017; Kaczkowski & Swartout, 2020; Kingori et al., 2018; Kumar et al., 2021; McMichael & Gifford, 2009). Increasing parental understanding could ease communication about sexual health (Dean et al., 2017; McMichael & Gifford, 2009) and make parents more approachable for questions on these topics (Kaczkowski & Swartout, 2020; Kingori et al., 2018; Kumar et al., 2021).

## Lines-of-Arguments Synthesis

Synthesizing the translations of the studies into themes, and reflecting on the themes, generated a lines-of-argument synthesis (Noblit & Hare, 1988). The synthesis illustrates how young refugees raise their voices and express a need for information from health professionals, despite the ubiquitous silence that represses them (see Supplemental File for a visual presentation of the Lines-of-Arguments synthesis).

The young refugees describe their experiences of the cultural and religious prohibitions that prevail around matters of sexual and reproductive health. Non-marital sex is considered stigmatizing, and abstinence is preached. Youth are risking grave consequences if they do not abide to cultural norms. They risk being kicked out of their home, being gossiped about, and judged by the community they belong to. In turn, this leads to feelings of shame and guilt. In a resettlement context these challenges are exacerbated. Their actions are more visible as they belong to small communities. The culture of silence that surrounds young refugees takes a new form, represented by language barriers, a lack of knowledge about sexual and reproductive health services, and laws that apply in a new country. The young refugees are raising their voices, expressing a desire for knowledge about sexual and reproductive health, not only for themselves, but for their parents as well. Nurses are vital in filling this knowledge gap in sensitive encounters, recognizing the double vulnerability of being a young person and a refugee, resettled in a new country.

## Discussion

This meta-ethnography has created a new understanding and knowledge of the perceptions refugee youth have on sexual and reproductive health, before and after resettlement to a new country. Their perceptions can aid nurses in delivering culturally competent care. In the *Culturally Competent Community care (CCCC) model* developed by Kim-Godwin et al. (2001, p. 919) culturally competent care consists of four dimensions, including caring, cultural sensitivity, cultural knowledge, and cultural skills.

A notable finding in the present study is that both cultural and religious values and contextual challenges in a new country contribute to, and maintain, the silence that surrounds young refugees regarding sexual and reproductive health. The silence deprives them of basic rights, presenting a barrier from receiving and accessing sexual health information, services, and care. The young refugees in the present study reported that not talking about sexual health fueled feelings of embarrassment and shame. The feeling of shame related to sexual health is also reported in other studies (Baroudi et al., 2021; Botfield et al., 2016, 2020; Ivanova et al., 2019; Lirios et al., 2024; Meldrum et al., 2016; Napier-Raman et al., 2023). Similarly other studies found that shame prevents adolescents from talking with their parents about sexual health (Ivanova et al., 2019; Lirios et al., 2024). Female participants in this meta-ethnography experienced that communication between mothers and daughters was superficial and limited to abstinence. A systematic review (Lirios et al., 2024) from Australia suggested that parental silence could be a means to protect family status and values. Other studies find that cultural and religious injunctions on abstinence before marriage discourage an open discussion related to sexual and reproductive health (Afroz et al., 2021; Lirios et al., 2024; Meldrum et al., 2016). Like our findings, a lack of discussion (Botfield et al., 2018), and stigma, makes it difficult for youth to access sexual health services and information (Meldrum et al., 2016; Napier-Raman et al., 2023). According to refugee youth in the present study the cultural communities they belong to in a resettlement context are small, and fear of being seen and judged by community members prevents them from seeking sexual and reproductive health services.

Our findings indicate that the silence and lack of communication and learning arenas is reflected in the level of knowledge about sexual and reproductive health. Several previous studies support our findings, suggesting that refugee youth have limited knowledge and misconceptions of sexual health (Baroudi et al., 2020; Botfield et al., 2018; Ivanova et al., 2019; Meldrum et al., 2016; Napier-Raman et al., 2023). Such as laws pertaining to sexual and reproductive health and rights (Botfield et al., 2020), condom use (Botfield et al., 2018), contraception, sexually transmitted infections (Ivanova et al., 2019; Lirios et al., 2024; Meldrum et al., 2016; Napier-Raman et al., 2023) and sexual and reproductive health services (Baroudi et al., 2020; Botfield et al., 2018, 2020; Lirios et al., 2024; Napier-Raman et al., 2023; Salehi et al., 2014). In our study we found that especially female refugees experienced negative emotions connected to the lack of knowledge of menstruation and sexual intercourse. Not being prepared brought fear, shock, and feelings of loneliness. Our findings further indicate that young refugees might experience being excluded from sexual health education at school because of language barriers. A Canadian study from 2014 (Salehi et al., 2014), also found that to a lesser extent newly arrived refugees received sexual health education. From a caring perspective, knowledge of the above is especially important for public health nurses, school nurses and nurses working in asylum centers, where sexual and reproductive health information and care is a significant part of the work assignment. To provide young refugees with knowledge can alleviate feelings of shame, fear of judgment and the mental stress of not knowing.

Some refugee youth in the present study shared that they have sexual relationships although sex before marriage is considered culturally unacceptable. Two Australian studies (Botfield et al., 2020; Napier-Raman et al., 2023) report that young refugees defy the cultural expectations of abstinence, although they are aware of the possibly harsh consequences of an unintended pregnancy. In our study refugee youth shared that a pregnancy could coerce young women into marriage, or they might risk being punished or kicked out of home. Lack of knowledge might lead to an unintended pregnancy and to the risk of sexually transmitted infections. In the present study we found that language barriers make it difficult to find out about, and access safe health services and to become familiar with laws on confidential services.

In the present study refugee youth shared that they would like to learn from health professionals with experience in the field of sexual and reproductive health. However, several also shared their concerns regarding confidentiality. This is also reported in several previous studies (Baroudi et al., 2021; Botfield et al., 2016; Lirios et al., 2024; Meldrum et al., 2016; Napier-Raman et al., 2023; Tirado et al., 2020). Like our findings, another study found that the use of interpreters heightened the fear that information would be disclosed (Botfield et al., 2016). Culturally competent care implies the importance of building trust by incorporating the client’s cultural system in their care (Kim-Godwin et al., 2001, p. 920). Kim-Godwin et al., 2001, p. 919) refers to the transcultural nursing scholar Madeleine Leininger who states that care is viewed negatively when it fails to fit with the population’s needs and expectations. In the CCCC model cultural sensitivity implies that nurses are aware of one’s own culture and that of the population being served, and that they acquire awareness and knowledge of issues that are important for planning health care services (Kim-Godwin et al., 2001, p. 922).

John Rawls’ Theory of Justice (Rawls, 2005) provided a framework for deepening the understanding of the synthesis. Rawls addresses justice as fairness, equal basic rights, and equality of opportunity. Young refugees are bearers of certain rights that cannot be disregarded, fairness is achieved when all have access to the information, services and care they need. Our findings indicate that young refugees face several barriers to attaining equal basic rights. Cultural and religious values and beliefs coupled with contextual barriers in a resettlement context, stand in the way of achieving an equality of opportunity, preventing young refugees from accessing sexual and reproductive health information, services, and care. The CCCC model underlines that caring also implies advocating for program development and lobbying at governmental level to create culturally meaningful health care policies (Kim-Godwin et al., 2001, p. 921). Young refugees in the present study express the need for sexual health information for themselves and their parents. Hence it is important for nurses to be aware of how cultural and contextual barriers influence the unique perspectives refugee youth have in relation to sexual and reproductive health, to fulfill their right and develop services that respect the diversity of the refugee youth population (2021, p. 922). The young refugees are asking for caring and non-judgmental health care professionals that can alleviate shame and embarrassment. In becoming culturally skilled nurses can build trust by strengthening the feeling of empowerment and decrease anxiety and fear, and thereby increase health care utilization among refugee youth (2021, p. 922).

### Strengths and Limitations

The strength of this meta-ethnography lies in the rigorous literature search, use of the CASP quality appraisal tool and transparency in following the seven-phase approach developed by Noblit and Hare (1988) and the eMERGe reporting guidance (France et al., 2019). The comprehensive searching was conducted in databases and other relevant sources (France et al., 2019). We judged that saturation seemed to have been accomplished when the same research papers appeared over again in databases and reference lists. The intention of this meta-ethnography has been to go beyond the aggregation of findings to interpret, integrate and create new knowledge (Bondas & Hall, 2007; France et al., 2019; Noblit & Hare, 1988). In the phase of analyses, all authors in the research team contributed by discussing and negotiating the themes and the overarching metaphor. The use of a metaphorical synthesis to visualize the interpretation of young refugees, who are breaking the silence with their secret voices, is considered a strength in meta-ethnography which can contribute to a deeper understanding of the phenomenon under study (Noblit & Hare, 1988). The aim of the study has been to explore the broader perception refugee youth have on sexual and reproductive health, and therefore articles with a specific study focus; sexually transmitted infections, sexual and gender-based violence, views on abortion, and contraception have deliberately been excluded. By excluding papers that contained information based solely on these or other singular topics, we might have missed valuable information that could have contributed to a richer or different understanding of the synthesis. Despite a comprehensive literature search, all relevant studies might not have been retrieved. Another limitation is that there were a limited number of studies conducted in Europe, Africa, Asia, and South America. By setting the limiter to English and Scandinavian articles, we might have missed relevant studies. Finally, conducting research on refugees possesses the challenge of presenting a superficial presentation. Refugees are a heterogenous group, in this meta-ethnography we have attempted to present the unique perception of 297 young refugees, originating from 13 countries. This is challenging to do within the scope of an article, without avoiding a degree of generalization. This is a major limitation and a potential blind spot.

## Conclusion

The lines-of-arguments synthesis in this meta-ethnography named by the overarching metaphor, *Secret voices are breaking the silence*, is a call that should be taken seriously by nurses and health organizations. The metaphors and the themes based on the translations express the need for focused and tailored attention on refugee youth and their need for sexual and reproductive health information, services, and care. The synthesized translations highlight that the young refugees in the studies not only experienced a silence concerning sexual and reproductive health within their own culture, but also in the country of resettlement. Nurses are in a position to help young refugees in breaking the silence. Young refugees need to receive culturally sensitive and equitable sexual health information services and care. Health interventions should be designed according to their needs, taking cultural considerations into account.

## Implications for Nursing Practice

This meta-ethnography contributes knowledge on refugee youth and their perception of sexual and reproductive health. Nurses and health organizations should address issues of trust and confidentiality by providing outreach sexual and reproductive health services, being aware of the social risk that is taken for young refugees when they are utilizing sexual health services, Sexual and reproductive health information should be given timely, providing young refugees with tools to decide freely on matters related to their sexual and reproductive health, and in accordance with their individual unique values. Caring also involves going beyond one’s professional responsibility, which implies addressing the legal and socio-economical barriers that stand in the way for receiving sexual and reproductive health information, services and care, and advocate for culturally meaningful health policies.

## Supplemental Material

sj-docx-1-gqn-10.1177_23333936251330688 – Supplemental material for Secret Voices are Breaking the Silence: A Meta-Ethnography of Perceptions of Sexual and Reproductive Health Among Resettled Refugee Youth Supplemental material, sj-docx-1-gqn-10.1177_23333936251330688 for Secret Voices are Breaking the Silence: A Meta-Ethnography of Perceptions of Sexual and Reproductive Health Among Resettled Refugee Youth by Tone Hjelm, Terese Bondas and Bente Kristin Høgmo in Global Qualitative Nursing Research

sj-docx-2-gqn-10.1177_23333936251330688 – Supplemental material for Secret Voices are Breaking the Silence: A Meta-Ethnography of Perceptions of Sexual and Reproductive Health Among Resettled Refugee Youth Supplemental material, sj-docx-2-gqn-10.1177_23333936251330688 for Secret Voices are Breaking the Silence: A Meta-Ethnography of Perceptions of Sexual and Reproductive Health Among Resettled Refugee Youth by Tone Hjelm, Terese Bondas and Bente Kristin Høgmo in Global Qualitative Nursing Research

sj-docx-3-gqn-10.1177_23333936251330688 – Supplemental material for Secret Voices are Breaking the Silence: A Meta-Ethnography of Perceptions of Sexual and Reproductive Health Among Resettled Refugee Youth Supplemental material, sj-docx-3-gqn-10.1177_23333936251330688 for Secret Voices are Breaking the Silence: A Meta-Ethnography of Perceptions of Sexual and Reproductive Health Among Resettled Refugee Youth by Tone Hjelm, Terese Bondas and Bente Kristin Høgmo in Global Qualitative Nursing Research

sj-docx-4-gqn-10.1177_23333936251330688 – Supplemental material for Secret Voices are Breaking the Silence: A Meta-Ethnography of Perceptions of Sexual and Reproductive Health Among Resettled Refugee Youth Supplemental material, sj-docx-4-gqn-10.1177_23333936251330688 for Secret Voices are Breaking the Silence: A Meta-Ethnography of Perceptions of Sexual and Reproductive Health Among Resettled Refugee Youth by Tone Hjelm, Terese Bondas and Bente Kristin Høgmo in Global Qualitative Nursing Research

## References

[bibr1-23333936251330688] Afroz T. Gele A. Thorsen V. C. (2021). Culture clash of female Somali adolescents and sexual and reproductive health services in Oslo, Norway. European Journal of Contraception & Reproductive Health Care, 26(4), 296–302. 10.1080/13625187.2021.1895109 33724125

[bibr2-23333936251330688] Aibangbee M. Micheal S. Mapedzahama V. Liamputtong P. Pithavadian R. Hossain Z. Mpofu E. Dune T. (2023). Migrant and refugee youth’s sexual and reproductive health and rights: A scoping review to inform policies and programs. International Journal of Public Health, 68, 1605801. 10.3389/ijph.2023.1605801 37342678 PMC10278890

[bibr3-23333936251330688] Baroudi M. Hurtig A.-K. Goicolea I. San Sebastian M. Jonzon R. Nkulu-Kalengayi F. K. (2021). Young migrants’ sexual rights in Sweden: A cross-sectional study. BMC Public Health, 21(1), 1618. 10.1186/s12889-021-11672-1 34482819 PMC8420038

[bibr4-23333936251330688] Baroudi M. Kalengayi F. N. Goicolea I. Jonzon R. Sebastian M. S. Hurtig A.-K. (2020). Access of migrant youths in Sweden to sexual and reproductive healthcare: A cross-sectional survey. International Journal of Health Policy and Management, 11(3), 287. 10.34172/ijhpm.2020.123 PMC9278465 32729283

[bibr5-23333936251330688] Bondas T. Hall E. Wikberg A. (2021). Metasynthesis and qualitative evidence in health care. In Liamputtong I. P. (Ed.), Research methods and evidence-based practice (4. utg), pp. 325–342. Oxford University Press. https://nla.gov.au/nla.obj-2995524286

[bibr6-23333936251330688] Bondas T. Hall E. O. (2007). Challenges in approaching metasynthesis research. Qualitative Health Research, 17(1), 113–121. 10.1177/1049732306295879 17170249

[bibr7-23333936251330688] Botfield J. R. Newman C. E. Bateson D. Haire B. Estoesta J. Forster C. Schulz Moore J. (2020). Young migrant and refugee people’s views on unintended pregnancy and abortion in Sydney. Health Sociology Review, 29(2), 195–210. 10.1080/14461242.2020.1764857 33411657

[bibr8-23333936251330688] Botfield J. R. Newman C. E. Zwi A. B. (2016). Young people from culturally diverse backgrounds and their use of services for sexual and reproductive health needs: A structured scoping review. Sexual Health, 13(1), 1–9. 10.1071/SH15090 26409644

[bibr9-23333936251330688] Botfield J. R. Zwi A. B. Rutherford A. Newman C. E. (2018). Learning about sex and relationships among migrant and refugee young people in Sydney, Australia: ‘I never got the talk about the birds and the bees’. Sex Education, 18(6), 1–720. 10.1080/14681811.2018.146490531275062

[bibr10-23333936251330688] Bukuluki P. Kisaakye P. Mwenyango H. Palattiyil G. (2021). Adolescent sexual behaviour in a refugee setting in Uganda. Reproductive Health, 18(1), 131. 10.1186/s12978-021-01181-0 34167555 PMC8222959

[bibr11-23333936251330688] Chandra-Mouli V. Svanemyr J. Amin A. Fogstad H. Say L. Girard F. Temmerman M. (2015). Twenty years after international conference on population and development: Where are we with adolescent sexual and reproductive health and rights? Journal of Adolescent Health, 56(1, Supplement), S1–S6. 10.1016/j.jadohealth.2014.09.015 25528975

[bibr12-23333936251330688] Dean J. Mitchell M. Stewart D. Debattista J. (2017). Intergenerational variation in sexual health attitudes and beliefs among Sudanese refugee communities in Australia. Culture Health & Sexuality, 19(1), 17–31. 10.1080/13691058.2016.1184316 27268405

[bibr13-23333936251330688] Dhar C. P. Kaflay D. Dowshen N. Miller V. A. Ginsburg K. R. Barg F. K. Yun K. (2017). Attitudes and beliefs pertaining to sexual and reproductive health among unmarried, female Bhutanese refugee youth in Philadelphia. Journal of Adolescent Health, 61(6), 791–794. 10.1016/j.jadohealth.2017.06.011 PMC5931208 28935387

[bibr14-23333936251330688] El Ayoubi L. L. Abdulrahim S. Sieverding M . (2021). Sexual and reproductive health information and experiences among Syrian refugee adolescent girls in Lebanon. Qualitative Health Research, 31(5), 983–998. 10.1177/1049732321989685 33733937

[bibr15-23333936251330688] France E. F. Cunningham M. Ring N. Uny I. Duncan E. A. S. Jepson R. G. Maxwell M. Roberts R. J. Turley R. L. Booth A. Britten N. Flemming K. Gallagher I. Garside R. Hannes K. Lewin S. Noblit G. W. Pope C. Thomas J. . . .Noyes J. (2019). Improving reporting of meta-ethnography: The eMERGe reporting guidance. BMC Medical Research Methodology, 19(1), 25. 10.1186/s12874-018-0600-0 30709371 PMC6359764

[bibr16-23333936251330688] Ivanova O. Rai M. Mlahagwa W. Tumuhairwe J. Bakuli A. Nyakato V. N. Kemigisha E. (2019). A cross-sectional mixed-methods study of sexual and reproductive health knowledge, experiences and access to services among refugee adolescent girls in the Nakivale refugee settlement, Uganda. Reproductive Health, 16(1), 35. 10.1186/s12978-019-0698-5 30890170 PMC6425697

[bibr17-23333936251330688] Kaczkowski W. Swartout K. M. (2020). Exploring gender differences in sexual and reproductive health literacy among young people from refugee backgrounds. Culture Health & Sexuality, 22(4), 369–384. 10.1080/13691058.2019.1601772 31032722

[bibr18-23333936251330688] Kim-Godwin Y. S. Clarke P. N. Barton L. (2001). A model for the delivery of culturally competent community care. Journal of Advanced Nursing, 35(6), 918–925. 10.1046/j.1365-2648.2001.01929.x 11555040

[bibr19-23333936251330688] Kingori C. Ice G. H. Hassan Q. Elmi A. Perko E. (2018). «if I went to my mom with that information, I’m dead»: Sexual health knowledge barriers among immigrant and refugee Somali young adults in Ohio. Ethnicity and Health, 23(3), 339–352. 10.1080/13557858.2016.1263285 27892706

[bibr20-23333936251330688] Korri R. Hess S. Froeschl G. Ivanova O. (2021). Sexual and reproductive health of Syrian refugee adolescent girls: A qualitative study using focus group discussions in an urban setting in Lebanon. Reproductive Health, 18(1), 1. 10.1186/s12978-021-01178-9 34167553 PMC8223310

[bibr21-23333936251330688] Kumar J. L. Chan W. Y. Spitz A. (2021). Pathways to sexual health among refugee young women: A contextual approach. Sexuality & Culture, 25(5), 1789–1807. 10.1007/s12119-021-09850-9

[bibr22-23333936251330688] Kwankye S. O. Richter S. Okeke-Ihejirika P. Gomma H. Obegu P. Salami B. (2021). A review of the literature on sexual and reproductive health of African migrant and refugee children. Reproductive Health, 18(1), 81–13. 10.1186/s12978-021-01138-3 PMC8052768 33865417

[bibr23-23333936251330688] Lirios A. Mullens A. B. Daken K. Moran C. Gu Z. Assefa Y. Dean J. A. (2024). Sexual and reproductive health literacy of culturally and linguistically diverse young people in Australia: A systematic review. Culture Health & Sexuality, 26(6), 790–807. 10.1080/13691058.2023.2256376 37755697

[bibr24-23333936251330688] Mason-Jones A. J. Nicholson P. (2018). Structural violence and marginalisation. The sexual and reproductive health experiences of separated young people on the move. A rapid review with relevance to the European humanitarian crisis. Public Health, 158, 156–162. 10.1016/j.puhe.2018.03.009 29653866

[bibr25-23333936251330688] McMichael C. Gifford S. (2009). “It is good to know Now. . .Before it’s too late”: Promoting sexual health literacy amongst resettled young people with refugee backgrounds. Sexuality & Culture, 13(4), 218–236. 10.1007/s12119-009-9055-0

[bibr26-23333936251330688] McMichael C. Gifford S. (2010). Narratives of sexual health risk and protection amongst young people from refugee backgrounds in Melbourne, Australia. Culture Health & Sexuality, 12(3), 263–277. 10.1080/13691050903359265 19904650

[bibr27-23333936251330688] Meldrum R. M. Liamputtong P. Wollersheim D. (2016). Sexual health knowledge and needs: Young Muslim women in Melbourne, Australia. International Journal of Health Services, 46(1), 124–140. 10.1177/0020731415615313 26536914

[bibr28-23333936251330688] Napier-Raman S. Hossain S. Z. Lee M.-J. Mpofu E. Liamputtong P. Dune T. (2023). Migrant and refugee youth perspectives on sexual and reproductive health and rights in Australia: A systematic review. Sexual Health, 20(1), 35–48. 10.1071/SH22081 36455882

[bibr29-23333936251330688] Noblit G. W. Hare R. D. (1988). Meta-ethnography: Synthesizing qualitative studies. Sage Publications.

[bibr30-23333936251330688] Ortiz-Echevarria L. Greeley M. Bawoke T. Zimmerman L. Robinson C. Schlecht J. (2017). Understanding the unique experiences, perspectives and sexual and reproductive health needs of very young adolescents: Somali refugees in Ethiopia. Conflict and Health, 11(1), 26. 10.1186/s13031-017-0129-6 29163667 PMC5688399

[bibr31-23333936251330688] Page M. J. McKenzie J. E. Bossuyt P. M. Boutron I. Hoffmann T. C. Mulrow C. D. Shamseer L. Tetzlaff J.M. Akl E.A. Brennan S.E. Chou R. Glanville J. Grimshaw J. Hróbjartsson A. Lalu M. M. Li T. Loder E. W. Mayo-Wilson E. Mcdonald S. … Moher D. (2021). The PRISMA 2020 statement: an updated guideline for reporting systematic reviews. bmj, 372. https://www.bmj.com/content/372/bmj.n71.short 10.1136/bmj.n71 PMC8005924 33782057

[bibr32-23333936251330688] Rawls J. (2005). En teori om retfærdighet [A Theory of Justice] ( Jacobsen M. C. , Overs.). DET lille FORLAG.

[bibr33-23333936251330688] Ruzibiza Y. Berckmoes L. Neema S. Reis R. (2021). Lost in freedom: Ambivalence on sexual freedom among Burundian adolescents living in the Nakivale refugee settlement, Uganda. Sexual and Reproductive Health Matters, 29(1), 232–245. 10.1080/26410397.2021.1889750 PMC8009026 33645469

[bibr34-23333936251330688] Salehi R. Hynie M. Flicker S. (2014). Factors associated with access to sexual health services among teens in Toronto: Does immigration matter? Journal of Immigrant and Minority Health, 16(4), 638–645. 10.1007/s10903-013-9961-y 24748054

[bibr35-23333936251330688] Starrs A. M. Ezeh A. C. Barker G. Basu A. Bertrand J. T. Blum R. Coll-Seck A. M. Grover A. Laski L. Roa M. Sathar Z. A. Say L. Serour G. I. Singh S. Stenberg K. Temmerman M. Biddlecom A. Popinchalk A. Summers C. . . .Ashford L. S. (2018). Accelerate progress—Sexual and reproductive health and rights for all: Report of the Guttmacher–Lancet Commission. Lancet, 391(10140), 2642–2692. 10.1016/S0140-6736(18)30293-9 29753597

[bibr36-23333936251330688] Tirado V. Chu J. Hanson C. Ekström A. M. Kågesten A. (2020). Barriers and facilitators for the sexual and reproductive health and rights of young people in refugee contexts globally: A scoping review. PLoS One, 15(7), e0236316. 10.1371/journal.pone.0236316 PMC7371179 32687519

[bibr37-23333936251330688] UNESCO, UN Women, UNICEF, UNFPA, & Joint United Nations Programme on HIV/AIDS. (2018). International technical guidance on sexuality education: An evidence-informed approach. Author. https://www.unwomen.org/en/digital-library/publications/2018/1/international-technical-guidance-on-sexuality-education

[bibr38-23333936251330688] UNHCR. (2023). UNHCR master glossary of terms. UNHCR. Available from: https://www.unhcr.org/glossary

[bibr39-23333936251330688] United Nations. (2024). Take action for the sustainable development goals. United Nations Sustainable Development. Available from: https://www.un.org/sustainabledevelopment/sustainable-development-goals/

[bibr40-23333936251330688] United Nations Population Fund. (2021, September 13). Comprehensive sexuality education. United Nations Population Fund. https://www.unfpa.org/comprehensive-sexuality-education

[bibr41-23333936251330688] World Health Organization. (2018). WHO recommendations on adolescent sexual and reproductive health and rights. World Health Organization. https://apps.who.int/iris/handle/10665/275374

[bibr42-23333936251330688] World Health Organization. (2023). Adolescent health. Adolescent Health in the South-East Asia Region. https://www.who.int/southeastasia/health-topics/adolescent-health

