# Supplementary material for: Secret Voices are Breaking the Silence: A Meta-Ethnography of Perceptions of Sexual and Reproductive Health Among Resettled Refugee Youth
Source: Glob Qual Nurs Res. 2025 Apr 30;12:23333936251330688. doi: 10.1177/23333936251330688 (PMC12044273; doi:10.1177/23333936251330688)
Supplement: sj-docx-1-gqn-10.1177_23333936251330688 – Supplemental material for Secret Voices are Breaking the Silence: A Meta-Ethnography of Perceptions of Sexual and Reproductive Health Among Resettled Refugee Youth [file sj-docx-1-gqn-10.1177_23333936251330688.docx]

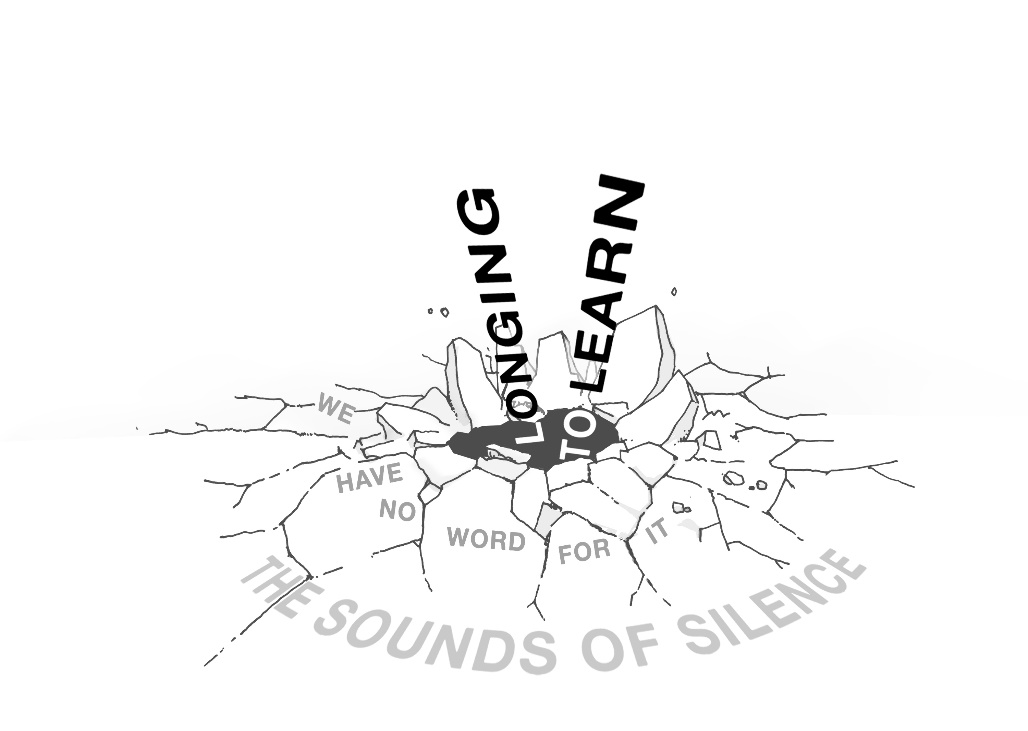


The lines-of-arguments synthesis: Young refugees’ perceptions of sexual and reproductive health: Secret voices are breaking the silence

The synthesis illustrates how young refugees raise their voices and express a need for information from health professionals, despite the ubiquitous silence that represses them. Their voices are expressed in the metaphor, “Secret voices are breaking the silence.” The silent water resembles *The sounds of silence*, which is covered by packed ice. In the darkness under the ice, there is no movement that indicates change. A culture of silenced shame and judgement has cemented the surroundings; it is reflected in the lack of communication that young refugees are experiencing concerning sexual and reproductive health. Due to cultural and religious values and beliefs that uphold the silence, and limited learning possibilities in a new country. They lack basic knowledge because they seem to have no one to talk to and nowhere to learn*.* Illustrated by *We have no words for it*, which is encapsulated in the ice, preserved by cultural and religious prohibitions and language barriers. The utterances from the young refugees’ secret voices are breaking the icy surface in a movement of *Longing to learn.*
