# Supplementary material for: Secret Voices are Breaking the Silence: A Meta-Ethnography of Perceptions of Sexual and Reproductive Health Among Resettled Refugee Youth
Source: Glob Qual Nurs Res. 2025 Apr 30;12:23333936251330688. doi: 10.1177/23333936251330688 (PMC12044273; doi:10.1177/23333936251330688)
Supplement: sj-docx-2-gqn-10.1177_23333936251330688 – Supplemental material for Secret Voices are Breaking the Silence: A Meta-Ethnography of Perceptions of Sexual and Reproductive Health Among Resettled Refugee Youth [file sj-docx-2-gqn-10.1177_23333936251330688.docx]

**Supplementary file**

**Table 4**. The translation process (examples).

| McMichael & Gifford (2009) ♀♂  Index paper | McMichael & Gifford (2010) ♀♂ | Dean et al. (2017) ♀♂ | Dhar et al. (2017) ♀ | Kingori et al. (2018) ♀♂ | Kaczkowski & Swartout (2019) ♀♂ | El Ayoubi et al. (2021) ♀ | Korri et al. (2021) ♀ | Kumar et al. (2021) ♀ | Translation | Subtheme | Theme |
| --- | --- | --- | --- | --- | --- | --- | --- | --- | --- | --- | --- |
| Concern about accessing specialist sexual health services, in case they are seen by a community member  Gossip spreads quickly | Young women who engaged in risky sexual activities were often described in derogatory terms | “If I got pregnant … that’s going to influence my Mum and Dad in the community” (female) | Sexual health risks are social risks  “When people came to know that she is taking pills, the community people, they just talk about her, and they will just laugh at her” | “We have fear that as a community, if we started discussing sexual health, we would be looked down upon” | Men also worried about their reputation with their parents and their community  Small community would see them | - | - | “How to go to a supermarket and ask for a condom. Actually, people don´t have that freedom”  [Fears] other people in the community discovering their use of sexual health services | Eyes and ears are everywhere | A culture of silenced shame | The sounds of silence |
| They [participants] referred to cultural and religious expectations of abstinence from sex prior to marriage | Narratives around protection emphasized prevention of pregnancy rather than STIs  Especially for women, abstinence demonstrates respect and loyalty to parents and maintenance of cultural values | Sex before marriage…was considered culturally unacceptable  “Oh no, you aren’t allowed…sex before marriage…its not ok” (female) | Premarital sex was stigmatized  "Yeah, in my opinion, like if we get pregnant before we get married, I feel like the boy think that I have already used the girl" | - | Learning about sexual health could potentially encourage sex before marriage, which would violate their sociocultural norms  "You do not need to learn about sex you don´t do it before marriage." (female) | Importance of abstinence before marriage was repeatedly emphasized | - | - | Abstinence is the rule | Sex is only a word until marriage |  |
| They don´t get taught about it.  "When you´re not going through school you miss that chance to learn about this kind of stuff"  Limited opportunities | “I never talked about it. This is my first time" | “ They don’t want their children to… know about sex…” (female) | - | No, I didn´t get any information from my mom … She just says beware of men." (female) | - | “we don´t talk about this with friends." | Complete and accurate information (…) was largely unavailable to them  Clear gaps in adolescents’ access to complete information | Lack of preparatory understanding | No arenas for learning | Nowhere to learn |  |
| Many participants indicated that they are reluctant or embarrassed to talk about sex with parents, doctors and peers | - | The embarrassing need to raise the topic with parents…who might disapprove | - | "I feel like in other communities, their parents can more easily talk to them about stuff and have the birds and the bees talk. We don´t get that." (female)  "We can´t have that talk with our parents and be comfortable with having that talk.”(female) | "You don´t want to get into that kind of conversation with your parents … you randomly ask a question, and they will take it down to whether you are interested in someone or not" (man) | Participants described several fears related to sexual health, including talking to their parents about having sex | Speaking with their mothers about sensitive topics can be embarrassing | Some of the participants talked about hiding their discovery from their parents for some time because of embarrassment … | Too embarrassed to approach parents | We can`t have that talk | We have no words for it |
| "And after getting married, then you have to do it, and you have no idea that you have to do this… It´s very different" (female) | - | - | - | - | - | - | Stark contrast between intimacy expectations and the details of sexual intercourse.  "He told me that [sex] will hurt and that I will bleed, but that it is normal … and that I shouldn´t be afraid" | - | To prepare for the unknown | What is to come is unknown |  |
| Strong desire to increase knowledge about sexual health  Energy of engagement with these often, sensitive issues  ” I would love to hear it ... I want to know"  "It´s good to know now, before you become active, and before it´s too late" | "I only know about HIV and AIDS that´s all. I don´t know the other virus and stuff. And I would like to know” | “They don’t have knowledge about the sexual diseases. They need to get educated about that” (female) | "…Actually, we have to learn about that boys and girls are equal. So, it´s better not to give up everything. We can do anything, what the boys can do"  "…So they talk about love, what is love…?” | “I wanted to know more “ (male) | They … expressed an interest in learning more | - | A period of considerable (anxiety) and curiosity | They showed interest in learning more.  Girls expressed their interest in having access to additional knowledge from experts in the field of SRH | Curious to know before it`s too late | A desire to learn | Longing to learn |
| The most accessible sources of information about SRH are through young people´s social networks. | - | Communication technology were identified as a useful source of sexual health information | SRH information was reportedly important for married women | - | Men felt comfortable discussing these topics with their peers ... With others who could easily relate to them  Advice from other people, such as friends, teachers or siblings | - | "When my sister got her period, I came to know about it. And when I got my period, I felt it was normal "  Adolescents sought support and information from each other | Others shared it immediately [first menstruation] with female members in order to get help | Information finds it`s way through different sources | Knowledge is to be found |  |
| - | - | - | - | Preferred receiving sexual health information from people they could confide in | "It´s all about making someone feel like it´s ok" (woman)  Supportive and knowledgeable staff would alleviate their [women’s] feelings of shame and embarrassment  Caring and non-judgmental staff | Doctors and nurses should be friendly, encouraging, and non-judgemental  "In order for youth to use sexual health services, she said that the clinic needs to "help them feel better, talk to them, and make them feel like this is a good thing"  Ensure that their staff are friendly | - | The exchange of knowledge should be done in a comfortable and private atmosphere with a trust-worthy person, who is direct and easy to talk to  They expressed their wish to be able to report the incident [sexual harassment] to an adult they trust and can freely talk to without being accused | It`s all about making someone fell like it’s ok | The young people are out there |  |
