# Supplementary material for: Secret Voices are Breaking the Silence: A Meta-Ethnography of Perceptions of Sexual and Reproductive Health Among Resettled Refugee Youth
Source: Glob Qual Nurs Res. 2025 Apr 30;12:23333936251330688. doi: 10.1177/23333936251330688 (PMC12044273; doi:10.1177/23333936251330688)
Supplement: sj-docx-3-gqn-10.1177_23333936251330688 – Supplemental material for Secret Voices are Breaking the Silence: A Meta-Ethnography of Perceptions of Sexual and Reproductive Health Among Resettled Refugee Youth [file sj-docx-3-gqn-10.1177_23333936251330688.docx]

**Supplementary file**

| **Table 5.** Translations to sub-themes, themes, and overarching metaphor. | |  |  |
| --- | --- | --- | --- |
|  |  |  |  |
| Translations (examples) | Sub-themes | Themes | Overarching metaphor |
| Eyes and ears are everywhere | A culture of silenced shame | The sounds of silence |  |
| It is an act against God |  |  |  |
|  |  |  |  |
| Abstinence is the rule | Sex is only a word until marriage |  |  |
| Opposing parents is risky |  |  |  |
|  |  |  |  |
| No arenas for learning | Nowhere to learn |  |  |
| Trusting the mother that does not know |  |  |  |
| Too embarrassed to approach parents | We can't have that talk | We have no words for it |  |
| Not seeking the help that is needed |  |  |  |
|  |  |  | Secret voices are breaking the silence |
|  |  |  |  |
| To prepare for the unknown | What is to come is unknown |  |  |
| You are opening the doors to sex |  |  |  |
| Curious to know before it`s too late | A desire to learn | Longing to learn |  |
| Learning the hard way |  |  |  |
|  |  |  |  |
| Information finds it`s way through different sources | Knowledge is to be found |  |  |
| School is the epicenter of knowledge |  |  |  |
|  |  |  |  |
| It's all about making someone feel like it's ok | The young people are out there |  |  |
| Make them understand |  |  |  |
