# Supplementary material for: Secret Voices are Breaking the Silence: A Meta-Ethnography of Perceptions of Sexual and Reproductive Health Among Resettled Refugee Youth
Source: Glob Qual Nurs Res. 2025 Apr 30;12:23333936251330688. doi: 10.1177/23333936251330688 (PMC12044273; doi:10.1177/23333936251330688)
Supplement: sj-docx-4-gqn-10.1177_23333936251330688 – Supplemental material for Secret Voices are Breaking the Silence: A Meta-Ethnography of Perceptions of Sexual and Reproductive Health Among Resettled Refugee Youth [file sj-docx-4-gqn-10.1177_23333936251330688.docx]

**Supplementary file**

**Table 6**. Context

| **Study** | **Young refugees and study characteristics** | **Country of origin** | **Country of origin and refugee camp: Vulnerabilities** | **Resettlement**  **country** | **Resettlement:**  **Vulnerabilities** |
| --- | --- | --- | --- | --- | --- |
| McMichael and Gifford (2009) | Recently arrived young humanitarian refugees, 67 males and 75 females (16-25 years)  14 interviews, 23 Focus groups, both ethnic and mixed  1-5 years in new country | Iraq, Afghanistan, Burma, Sudan, Liberia, The Horn of Africa | Instability of life in a refugee camp and lack of sexual health education in the country of origin. | Metropolitan area, Melbourne, Australia | Forced migration, displacement, resettlement issues and competing demands. Few sources for sexual health knowledge in use. Confidentiality issues, shame and embarrassment when searching for knowledge. Risk of falling in between health services. |
| McMichael and Gifford (2010) | Recently arrived young humanitarian refugees, 67 males and 75 females (16-25 years)  14 interviews, 23 Focus groups, both ethnic and mixed  1-5 years in new country | Iraq, Afghanistan, Burma, Sudan, Liberia, The Horn of Africa | Disrupted education, limited health care access, fragmentized social and family networks, violence, persecution and insecurity. Lack of focus on sexual health promotion in country of origin and refugee camps. High risk of STI and HIV | Metropolitan area, Melbourne, Australia | Family disruptions, new languages, unfamiliar education, new housing, health and legal issues,  changing religious, moral and cultural values, including sexuality. |
| Dean et al. (2017) | Recently arrived Sudanese humanitarian young refugees.  5 male and 6 female (19-24 years).  4-8 years in new country | Sudan | Traditional family and parenthood’s right to decide on the young person’s behavior.  Moral and religious values. Keeping secrets from parents. | Queensland, Australia | Intergenerational discord: Beliefs and patterns of sexual behavior, relationships and parenthood vs young person’s rights. Parenthood losing control over the young person. |
| Korri et al. (2021) | Married and unmarried Kurdish and Arab Syrian adolescent girls, aged between 13 and 17 years.  The sample size of the study,  consisted of 40 participants in eight FGDs. | Syria | Adolescent refugee girls exposed to precarious conditions, more prone to sexual and reproductive health problems. | North-East  Beirut | Sexual harassment by fellow male refugee, poor knowledge about female reproductive system, being under ‘social surveillance’ once reached puberty. A wish to be able to speak to a specialized practitioner about puberty, menstruation, and female physiology. |
| El Ayoubi et al. (2021) | (a) 14- to 17-year-old unmarried Syrian adolescent girls; (b) 15- to 20-year-old married Syrian  adolescents who married before age 18. | Syria | High rates of early marriage, expected to get pregnant. Refugee girls experience barriers to education. Syrian refugees  disadvantaged given the neglect of their needs in local responses to the Syrian humanitarian crisis | Lebanon | *Shocked* and *scared* upon the sight of the  blood of their first period. Lack of knowledge about sex brought fear, worry, and loneliness on wedding night. Social norms contributed to a generalized silence and a delay in relaying information |
| Dhar et al. (2017) | 14 female, Bhutanese refugees, ages 16-20 years, never married, and never pregnant.  Individual interviews.  1-4 years in new country. | Bhutan (born  in refugee camps in Nepal, resettled in the U.S. from 2011 to 2014) | Variable knowledge and educational possibilities. Cultural and religious values. Young women vulnerable to coercion into marriage if pregnant.  Arranged marriages. Gender discrimination. | Philadelphia, USA | Not comfortable seeking SRH information or using hormonal contraception as premarital sex was stigmatized. Not included in SRH education because of language. Misconceptions (access to SRH services, need consent from partner to have an abortion). Misunderstanding legal rights. |
| Kingori et al. (2018) | Somali young adults aged 18–25 years, 14 females, 13 males.  In-depth interviews  41% relocated to the US between 0-5 years old. | Somalia | Societal norms perpetuated a culture of silence. Religious barriers to health knowledge and contraception use. | Ohio, USA | Prevented from receiving sexual and reproductive health education by parents and religious leaders. Got inaccurate information. Judgement from community members, fear of being ostracized. |
| Kaczkowski and Swartout (2019) | Newly resettled refugee youth aged 18-24 years, unmarried, no children, 12 women, 13 men.  Three focus groups with men and 12 interviews with women.  1-4 years in a new country. | Afghanistan, Burma/Myanmar, Central African Republic, Colombia, the Democratic Republic of Congo, Pakistan, Somalia | Variable knowledge and educational possibilities. Cultural norms an restrictions. | Atlanta, USA | Little knowledge of health services and age limits. Misconceptions about sexually transmitted infections and contraception. Secret relationships. Financial burdens, language barriers. Personal barriers (embarrassment and confidentiality). Members of small communities, scared of being seen. |
| Kumar et al. (2021) | 12 female refugees ages 18-24.  Individual interviews.  Most lived in the US for over 5 years. | Burma, Central African  Republic, the Democratic Republic of Congo, and Somalia | Sociocultural restrictions (parents, religion and culture) regarding sex and relationships. | Large southeastern city, USA | Judgement from family and peers and their community. Including judgment from American friends/society. Difficult to fit in. Judgement preventing them in using sexual health services. Personal barriers. Judgment-fear-embarrassment linked to shame/guilt: negative consequences for well-being (suicide, unhealthy behaviors). |
